# Authors’ Response to Peer Reviews of “Beyond Expected Patterns in Insulin Needs of People With Type 1 Diabetes: Temporal Analysis of Automated Insulin Delivery Data”

**DOI:** 10.2196/66643

**Published:** 2024-11-27

**Authors:** Isabella Degen, Kate Robson Brown, Henry W J Reeve, Zahraa S Abdallah

**Affiliations:** 1 Interactive Artificial Intelligence Centre for Doctoral Training, School of Computer Science, Faculty of Science and Engineering, University of Bristol, 1 Cathedral Square, College Green, Bristol, BS1 5DD, United Kingdom, 44 7726100905; 2 University College Dublin President's Office, College of Engineering and Architecture, University College Dublin, Dublin, Ireland; 3 School of Mathematics, Faculty of Science and Engineering, University of Bristol, Bristol, United Kingdom; 4 School of Engineering Mathematics and Technology, Faculty of Science and Engineering, University of Bristol, Bristol, United Kingdom

**Keywords:** multivariate time series, k-means, clustering, machine learning, temporal patterns, data-driven, openAPS, open dataset, type 1 diabetes, insulin needs

*This is the authors’ response to the peer-review reports for “Beyond Expected Patterns in Insulin Needs of People With Type 1 Diabetes: Temporal Analysis of Automated Insulin Delivery Data.”*

## Round 1 Review

### Anonymous [1]

#### General Comments

*Degen et al [* *2* *] present the results of time series data derived from the OpenAPS data commons. The paper represents an important contribution to the field of diabetes technology research, as most of the work so far focused on clinical outcome analysis only. Pattern analysis of the device data provides useful insights for the entire open science community around open-source automated insulin delivery (AID) and will help researchers to identify their next research questions.*

*There are few papers on temporal patterns in AID research, which is why I support the publication of this comprehensive and well-written report.*

Response: We would like to thank the reviewer for this excellent summary and their support for our work despite having submitted it in a poor format for JMIR. We hope that the changes made have further strengthened the paper and made it more useful for the open science community and their research.

#### Specific Comments

*1. Did the authors analyze any demographics from participants? This would be essential to exclude selection bias or at least highlight limitations if the sample is not representative of most users of open-source AID.*

Response: The first version of the paper was presented at the Time Series for Health workshop at the NeurIPS 23 conference; for this, we didn’t analyze the demographic data for participants. Recognizing how important this is for any JMIR journal, we have analyzed the demographic data now and added the results in the second version of the paper. The decision on whose data to analyze was solely made based on the amount of data that had been submitted by each person (see flowchart in Figure 1). To be able to analyze patterns in days, we decided to only look at people who had at least 30 days of data with at least one recording of insulin on board (IOB), carbohydrates on board (COB), and interstitial glucose (IG) every hour (n=29). Furthermore, we only looked at the same subtype of the OpenAPS system: the OpenAPS, not AndroidAPS or Loop. The OpenAPS system has more individuals (n=116) compared to the other two systems. This was both a practical decision with regard to preprocessing the data due to the device log files being different between the systems, but this also means that we are reporting on the same system and therefore avoid comparing patterns that perhaps arise between the different systems. For the 29 people, 26 had some or all of the self-reported demographics supplied. We have made this selection clearer in the Methods, and we’ve added the limitations around the data.

*2. How many individual datasets were analyzed, and what is their total time span?*

Response: Following on from the response to the first comment, we preprocessed the data for 116 participants and analyzed 29 in detail. We’ve hopefully made this much clearer with the changes made to the Methods and Results sections. The amount of data submitted by each individual varies greatly, and we want to make sure that the patterns found are not just one odd day here and there. This is the reason why we limited the detailed pattern analysis to 29 datasets for whom we had at least 30 days of data. For these 29 participants, the time series data spans, in mean values, 1.62 (SD 0.6) different years, 6.38 (SD 3.7) different months, and 80.14 (SD 75.3) different days. This information has also been added to the Results section, see Table 2. The variation in the amount of data even across the subset of individuals with at least 30 days of data remains bigger than we wish. This is one of the reasons why we use 95% CIs of mean values to establish significant patterns, taking into account the hugely varying number of samples across individuals for different units of time. Furthermore, we have added the new results and presented the frequency of patterns for 29 people. Hopefully, this has significantly improved our paper.

*3. I suggest using “interstitial glucose” or “sensor glucose” versus blood glucose as continuous glucose monitoring sensors are usually placed subcutaneously and therefore do not measure (capillary) blood glucose.*

Response: Thank you for this comment. In the second version of the paper, we now use the abbreviation “IG” for interstitial glucose, which is much more precise about what is measured.

### Reviewer CH [3]

#### General Comments

*This paper presents a study about temporal patterns in insulin needs for type 1 diabetes (T1D). It employs various time series techniques to spot such patterns using matrix profiles and multivariate clustering. The OpenAPS Data Commons dataset, an extensive dataset collected in real-life conditions, was analyzed to discover temporal patterns in insulin need driven by well-known factors such as carbohydrates and potentially novel factors. The results are limited to disclosing interesting temporal patterns in insulin need that cannot be explained solely by carbohydrates through the performed analysis. While patterns found are auspicious, they still lack scientific rigor and research into correlations and causalities that drive these patterns to truly inspire new research into T1D.*

Response: Thank you for your thoughtful comments and feedback. We have made extensive changes to the paper submitted and hope that the second version of the manuscript addresses your comments. In particular, we have more thoroughly analyzed the patterns found across the individuals, given more information about the demographics of the individuals and other characteristics, and compared the frequency of patterns that cannot be solely explained by carbohydrates to those that can.

#### Specific Comments

##### Major Comments

*1. The research is well-designed and developed.*

Response: Thank you. We hope this continues to hold for the second version of the manuscript. Please, note that we’ve made significant changes. Most importantly we have been clearer and more consistent about the people selected for analysis and have included their demographics where available. However, this has also meant that for space and conciseness, we’ve dropped the results from the matrix profile analysis of weeks due to them not highlighting any new results. We, however, do refer back to first version of the manuscript, which was the original NeurIPS workshop paper that still contains these results. We hope you find our additional methods to analyze the frequency of patterns across the different people now included in the second version of the manuscript more scientifically rigorous. We’ve also considerably expanded the Discussion section. There are still limitations, and we’ve added a section on these in the discussion.

*2. Although the paper is well written and presents interesting results, it does not comply with the Instructions for Authors of JMIR [4].*

Response: We would like to apologize for that. The paper was written for the TS4H Workshop at NeurIPS 22 and was never restructured to follow the JMIR style. We have extensively reworked the paper and hope it is now compliant.

*3. The discussion and implications are minimal, leaving a more significant contribution to future work.*

Response: This is a fair point and thank you for making it. As mentioned earlier we have extended the analysis, presented new results, and completely rewritten the Discussion section. We’ve also been clearer about the limitations. We hope that readers will take away that unexpected patterns happen and they happen frequently and warrant further research into the factors driving them. We’re curious to hear if the second version of the manuscript improves this point at least to some extent.

### Anonymous [5]

*The Word file containing the manuscript is encoded with a mess and not readable.*

Response: We’d like to apologize for the messy file. We’ve completely reworked the Word file. We hope the second version of the manuscript is readable.

## Round 2 Review

### Anonymous [5]

*This paper presents a comprehensive analysis of insulin needs in people with T1D using AID data. The study aims to uncover unexpected temporal patterns in changes in insulin needs, which could potentially offer new insights into T1D understanding and treatment. The research design and methodology are well-structured and makes use of a wide range of statistical and machine learning tools.*

*There are a few areas that could be improved or clarified, below are my comments:*

Response: We would like to thank the reviewer for their valuable feedback on our manuscript. We believe that the suggested changes have strengthened our paper. We address each comment made in detail below.

*1. The study is based on data from 29 individuals. What is the generalizability of the results and conclusions drawn from the analysis? How did the authors ensure the statistical power of the study and make sure the findings are applicable to other cohorts?*

Response: Thank you for asking us these questions. They have prompted us to make significant changes that we believe improve our study. We now not only report on *P* values like we did in revision 2 but also on standardized empirical effect sizes to provide better information about how strong or weak the differences in means in the various analyses are (see Frequency of Patterns, Relationships and Comparison sections in the Methods and Results and Table 5). To aid effect size analysis and statistical interpretation when comparing the CIs of mean values with each other, we now compare the CIs for the difference in mean values between the groups with each other. The version of the OpenAPS Data Commons dataset used in our study has a small number of participants with at least 1 month of data (n≤29, depending on the time granularity). On top of that, the amount of data contributed per participant is not consistent (see Data and Population in the Methods and Results and updated Limitations). This is not ideal and does reduce the ability to discover patterns. However, our study is an exploratory study, and our hypothesis is to discover if unexpected patterns even exist. Our aim is not to accurately define the frequency or analyze causes for these patterns (we simply do not have the data to do that) but to demonstrate that these patterns exist and that it is worth studying them further. To be able to achieve this, we have taken great care to use conservative statistical methods with a focus on reducing type 1 errors (in our context the discovery of patterns where there are none) at the cost of making more type 2 errors (not identifying a pattern where there is one). Given that our main conclusion is that “unexpected patterns exist and that there are as common as expected ones,” we feel this is the right approach, and we are confident that similar findings can be achieved for other AID datasets. To strengthen this, we have now also introduced Bonferroni to control the family-wise error rate at 5% when comparing multiple means (eg, the means of all the hours of the day). This means for identifying significant differences in means for hours of the day, we have reduced the α from .05 to .0002, for the clusters to .0021, for days of the week to .0024, and for months of the year to .0008. Where we compare the differences of the overall means between the clusters weekdays and weekends, the α was reduced to .0018, for comparing summer and winter months to .0063, and for comparing first and second year use of AID to .0032. These changes have reduced the frequency of patterns we report (see changes to Table 4), but they have not impacted the conclusions we can draw from these numbers. As requested, we now also report for which effect sizes we achieve a power of 80% as well as what number of sample participants would be required for a power of 80%. It is important to note that the patterns are determined per participant, and we then conclude the frequency across participants from that. For each participant, we do have at least 1 month of data. The mean number of hours of data for all participants is 1923 hours, see Table 2. We purposefully reduced the number of participants we included to ensure that we had a reasonable amount of data for each participant at the cost of having fewer participants. For the patterns in hours of the day and in clusters, the mean effect sizes are large (*d*>0.94), for days of the weeks and months of the years, the effect sizes are smaller (0.3<*d*<0.52). Despite using Bonferroni-adjusted α, the power for over 50% of detected patterns remains ≥80%, see newly added Table 5. Only having 29 participants plays the biggest role when comparing the overall IOB, COB, and IG to ascertain if we can find a significant difference in mean values between the clusters, weekdays and weekends, winter and summer months, and first and second year of data donated. We have not found significant differences and make no conclusions from these tests other than that we cannot find significant differences. The power analysis tells us that for these analyses, we would need at least 44 participants for a power of 80% for the clusters, and for the weekdays and weekends, we would need >13,000 participants (effect sizes are small for IOB, which further supports that these differences likely don’t exist), and for winter and summer months, we would need 88 participants. We have added the results of the power analysis. We hope that reporting on the effect sizes and power helps the reader form their judgment about generalizability and helps researchers with the design of future studies on this topic.

There is a second angle to your question regarding comparison with other cohorts. Our research is only possible using high-resolution data automatically collected by AID devices (that excludes manually logged data for multiple daily injection treatment). Additionally, to be able to discover unexpected patterns, we rely on the AID automatically adjusting insulin. This does not happen in multiple daily injection treatment and therefore would make it hard to distinguish between human dosing error and an unexpected pattern. We have hopefully made this clearer in the introduction. Our results show higher effect sizes when looking at time granularities with higher resolution (eg, hours instead of months or years). In the results for data and population, we report how the demographic data of our participants compare to national data for T1D, and we found that our cohort in comparison has lower hemoglobin A_1c_ levels and has been using advanced technologies for treating T1D for longer than the national standards for T1D suggest. We conclude that our cohort are early T1D technology adopters. In our relationship analysis (see our response below), we discovered that the frequency of patterns E1/E3 (higher IOB is needed for higher COB) are related to the amount of carbohydrates being eaten, the frequency of pattern U2 (higher IG is not due to higher COB) is related to having used an insulin pump for less long, and the last laboratory-reported hemoglobin A_1c_ level too is both positively and negatively associated with expected and unexpected patterns. Based on this, we can conclude that hemoglobin A_1c_ level, the amount of carbohydrates eaten, and how long an insulin pump has been used influence the frequency of patterns. It remains to be seen if a cohort with less technology experience and higher hemoglobin A_1c_ levels would still have the patterns or not, and we mention this in our conclusion. We have no evidence that these patterns do not happen in all people with T1D and believe they do, but this remains to be proven in future research.

*2. The paper primarily focuses on identifying patterns in the insulin needs of people with T1D. However, it doesn’t clearly outline how these patterns could be used to predict future insulin needs. A predictive validation of the identified patterns could strengthen the study.*

Response: Thank you for asking us to clarify how these patterns could be used to predict future insulin needs. Our paper aims to discover expected and unexpected temporal patterns in changes in insulin needs in data from AID systems, analyze how common they are, and investigate if we can relate them to demographic information or other characteristics. Our aim for this study is not to predict insulin needs, we sadly do not have the data on factors that drive insulin needs beyond carbohydrates. Our results highlight that predicting IG from IOB and COB is challenging as Granger causality is not consistent. We hope that our results instead inspire more research into lesser explored factors that drive changes in insulin needs and lead to these factors being measured, quantified, and fed back into insulin timing and dosing decision-making and eventually could be used for predictions. Hopefully, the changes we’ve made to the introduction including a discussion of the confounding factors (see below) and clarifying practical applications (see below) as well as a reorganization of the methods and results (see below) and cleaning up of the Discussion with regards to all of these changes have made it clearer what role these patterns play and why we study them.

*3. Too many unnecessary bullet points and bold text in the paper, which significantly hinders easy reading and understanding.*

Response: Thank you for this fair critique and for inspiring us to reorganize the Methods and Results. As you have pointed out in revision 2, our Methods and Results sections made use of many bullet points and bold text. We have completely reorganized the subsections in the Methods and Results. In revision 2, we had two subsections in the Methods and Results: Data and Population and Pattern Discovery (and many bold sections). In revision 3, we now have reorganized the Methods and Results into the following subsections: Data and Population (unchanged); Frequency of Expected and Unexpected Patterns (existing analysis but extended); Relationships Between Pattern Frequency and Factors (new analysis to address feedback below); Comparison of IOB, COB, and IG (existing analysis but extended to be complete); and Forecastability of IOB, COB, and IG (existing analysis). As this represents the importance of the results, we have used this same organization for the principal results in the Discussion. We hope these changes make it easier to read and understand our paper.

*4. The study acknowledges that factors beyond carbohydrates might influence insulin needs, but it doesn’t delve into what these factors might be. There could be confounding factors such as physical activity, stress, illness, etc, which might have influenced the insulin needs of the participants. The author needs to provide an analysis or discussion of these factors.*

Response: Thank you for this feedback. We have rephrased the paragraph in the introduction that describes how insulin dosing decisions are made and have added a description of a few important confounding factors. We also clarified throughout the manuscript that we believe such confounding factors cause the unexpected patterns that we discover and describe in Table 1. We are sorry that this was not clear in revision 2. Given that we do not have data about any of these confounding factors, we sadly cannot do any causal analysis of them. We instead highlight how frequent unexpected patterns are, show the times when we believe these factors are at play in AID data, and explain why we think AID data is suited to study such factors. We hope our research will inspire more research and result in more AID datasets being made available with high-frequency data that include sensor information from more confounding factors such as activity levels, stress, body temperature, etc.

*5. Factors such as age, sex, or duration of diabetes can very likely influence insulin needs. Can the authors add some additional analysis around these factors? I suspect this could provide additional insights and potentially reveal more patterns.*

Response: Thank you for this great suggestion. It helped us link the demographic data with the pattern discovery work. We have now investigated the relationships between the frequency of the patterns and the demographic factors as well as factors from the AID device. We describe this in the Methods and Results subsection Relationships Between Pattern Frequency and Factors. In short, we investigated Kendall τ associations between the frequency of each pattern E1-E3 and U1-U2 for each participant in each time granularity (hours of the day, clusters, days of the week, months of the year) with the demographic factors (age, duration of T1D, last laboratory-reported hemoglobin A_1c_ level, average carbohydrates, average insulin, average basal insulin, pumping since, using continous glucose monitoring since, and using AID since) and AID device data (number of hours; number of days; number of months; number of years; and overall mean IOB, COB, and IG). Sadly, we do not have sufficient data to investigate associations with sex as most participants did not reveal their sex, and only 2 of those that did were female. We have also updated our principal results with a summary of these findings.

*6. It would be beneficial if the authors compared their approach with at least one existing method for analyzing insulin needs in patients with T1D. This would allow for a better understanding of the advantages and limitations of their method.*

Response: Thank you for pointing out that we have indeed failed to describe how insulin needs are commonly analyzed in people with T1D. We have added a brief description of eating and fasting experiments that are commonly performed in clinical practice to determine how well insulin needs are met and to adjust treatment accordingly. We also explicitly link to Dose Adjustment For Normal Eating (DAFNE) as it provides a solid base of literature around adjusting insulin and its efficacy and shortfalls. We would also like to clarify that the intent of our method is not to provide a new method for determining insulin needs in clinical practice. We intend to highlight the impact that factors beyond carbohydrates have on insulin needs. We use existing methods to analyze expected and unexpected patterns of insulin needs in AID data. Our goal is to highlight, that despite our participants having excellent outcomes (hemoglobin A_1c_ level mean 46 mmol/mol, see Table 2), the AID system frequently adjusts insulin in unexpected ways to keep IG in that tight range and highlight the importance of considering the impact of confounding factors (see our response above). We hope our changes to the Introduction have made this clearer.

*7. I suggest a more in-depth discussion on the findings, especially focus on how it could be practically applied in the management and treatment of T1D. For instance, how could these patterns help in developing more effective AID systems or in informing patient education and self-management strategies?*

Response: Thank you for motivating us to connect our findings to clinical practice. While we are currently in the research (not translational) phase, the bigger goal of our research is to eventually be able to contribute to improving the treatment of T1D. Where we describe the aim of our study in the Introduction, we have now clarified that we are analyzing AID data for expected and unexpected factors because we believe that measuring and quantifying the impact of factors beyond carbohydrates is important for better insulin dosing and timing decision-making. We also mention that in the case of AID treatment such information could be automatically fed back to the algorithm to adjust insulin more timely. However, in both cases, we need to understand the impact of these factors on different people with T1D better before adjusting clinical practice. We mention this in our Conclusion.

*8. The study’s reliance on self-reported data might introduce bias or inaccuracies, as this data is subject to memory recall and honesty of the participants. I suggest stating this limitation in the Discussion.*

Response: Thank you for raising this important concern. Your question has inspired us to add a section in Limitations with potential biases such as selection volunteer bias, technology bias, and demographic bias of the participants. We hope this addresses your concerns. We would also like to clarify that our study does not rely on self-reported data, and we have made this clearer in the manuscript. The data we analyze for patterns is data automatically recorded by the AID system (it stems from logs of the device). This data does not suffer from recall issues, and the honesty of the participants does not impact its quality, which on top of the much higher frequency of logging is another strength of studying AID data. We have clarified this in the Introduction where we describe AIDs. The only data that is self-reported is the demographics data, which we use to describe our cohort (see Methods and Results: Data and Population) and for relationship analysis (to address your feedback above). This, however, does not affect our pattern discovery. It merely impacts/limits potential explanations for these patterns, which is not the main focus of our study. The contribution of the AID data and the demographic data are made on a voluntary basis. Nobody was forced to donate any data. We believe any mistakes in the demographic data are honest mistakes, free from intentional deception. For us, this is evident also in the fact that demographic data is missing. We can conjecture it is missing because either participants did not bother to provide demographic information, could not remember some of the information, or have not felt comfortable disclosing all of their information, which sadly seems to be particularly true for disclosing sex. Figure 1 and Table 2 describe how many participants have which demographic data. The selection of the participants we studied to discover patterns is not based on their demographic data. It is based alone on how much data a participant has donated to the OpenAPS Data Commons (see Methods: Data and Population). To address the above feedback, we have also extended the limitations around AID data.

## Abbreviations

AID: automated insulin delivery
COB: carbohydrates on board
DAFNE: Dose Adjustment For Normal Eating
IG: interstitial glucose
IOB: insulin on board
T1D: type 1 diabetes

## References

[1] Anonymous (2024). Peer review of “Beyond Expected Patterns in Insulin Needs of People With Type 1 Diabetes: Temporal Analysis of Automated Insulin Delivery Data”. JMIRx Med.

[2] Degen I, Robson Brown K, Reeve HWJ, Abdallah ZS (2024). Beyond expected patterns in insulin needs of people with type 1 diabetes: temporal analysis of automated insulin delivery data. JMIRx Med.

[3] Carvalho D (2024). Peer review of “Beyond Expected Patterns in Insulin Needs of People With Type 1 Diabetes: Temporal Analysis of Automated Insulin Delivery Data”. JMIRx Med.

[4] Instructions for authors of JMIR. JMIR.

[5] Anonymous (2024). Peer review of “Beyond Expected Patterns in Insulin Needs of People With Type 1 Diabetes: Temporal Analysis of Automated Insulin Delivery Data”. JMIRx Med.

